# New Molecular Mechanism of Superbiofilm Elaboration in a Staphylococcus aureus Clinical Strain

**DOI:** 10.1128/spectrum.04425-22

**Published:** 2023-01-31

**Authors:** Liansheng Yu, Junzo Hisatsune, Shoko Kutsuno, Motoyuki Sugai

**Affiliations:** a Antimicrobial Resistance Research Centre, National Institute of Infectious Diseases, Tokyo, Japan; b Project Research Center for Nosocomial Infectious Diseases, Hiroshima University, Hiroshima, Japan; c Department of Antimicrobial Resistance, Hiroshima University Graduate School of Biomedical & Health Sciences, Hiroshima University, Hiroshima, Japan; Institut Necker Enfants Malades

**Keywords:** *Staphylococcus aureus*, biofilm, transcriptional repressor, *icaR*

## Abstract

Previously, we reported a novel regulator of biofilm (*rob*) with a nonsense mutation in the superbiofilm-elaborating strain JP080. Intriguingly, the complementation of JP080 with wild-type *rob* did not completely abolish its superbiofilm-elaborating phenotype. Therefore, we searched for other possible mutation(s) using complete genome sequence data and found a missense mutation in the gene *icaR*, which altered its 35th amino acid (Ala35Thr). To further study the mechanism of superbiofilm elaboration in JP080, we reconstructed the same mutations of *rob* and *icaR* in the strain FK300 and analyzed the phenotypes. The mutation of *rob* (A331T) increased biofilm elaboration, as previously demonstrated; similarly, an *icaR* mutation increased poly-*N*-acetylglucosamine and biofilm production in strain FK300. Furthermore, our analyses indicated that the double mutant of *rob* and *icaR* produced significantly more biofilms than the single mutants. Additionally, gel shift analysis revealed that the *icaR* from JP080 lost its ability to bind to the *ica* promoter region. These findings suggest that the *icaR* mutation in JP080 may result in a nonfunctional protein. We compared *ica* operon expression in an *icaR* single mutant, *rob* single mutant, and *rob* and *icaR* double mutant to the wild type. The *rob* and *icaR* mutants showed increased *ica* operon transcription by approximately 19- and 79-fold, respectively. However, the *rob* and *icaR* double mutant showed an approximately 350-fold increase, indicating the synergistic effects of *icaR* and *rob* on JP080 biofilm elaboration. Consequently, we concluded that the double mutations *rob* and *icaR* synergistically increased *ica* operon transcription, resulting in a superbiofilm phenotype in Staphylococcus aureus.

**IMPORTANCE** Poly-*N*-acetylglucosamine (PNAG) is a major component of S. aureus biofilm. PNAG production is mediated by the products of four genes, *icaADBC* encoded in the *ica* operon, and the major negative regulator of this operon is IcaR encoded just upstream of *icaADBC*. Previously, we reported another negative regulator, Rob, through gene expression analysis of clinically isolated superbiofilm-elaborating strain JP080. The *rob* gene is encoded at different loci distant from the *ica* operon. Here, we report that JP080 also carried a mutation in *icaR* and demonstrated that IcaR and Rob synergistically regulate PNAG production. We successfully reconstructed these mutations in a wild type, and the double mutant resulted in superbiofilm-elaborating phenotype. We clearly show that loss of function of both IcaR and Rob is the very reason that JP080 is showing the superbiofilm-elaborating phenotype. This study clearly demonstrated there are at least two independent regulators synergistically fine-tuning PNAG production and suggested the complex regulatory mechanism of biofilm production.

## INTRODUCTION

Staphylococcus aureus is an opportunistic bacterial pathogen that causes many infections, from superficial skin and mucosal infections to bone or lung infections and serious systemic diseases. Longitudinal studies of S. aureus colonization showed that approximately 20% (range, 12 to 30%) of individuals are persistent S. aureus carriers, and 30% (range, 16 to 70%) are intermittent carriers (1). Colonization plays a key role in S. aureus transmission and is a known risk factor for the subsequent development of infections. Biofilms protect bacteria from host immune responses and antibiotics (2). The biofilm matrix is a physical barrier composed mainly of polysaccharides, cell surface and secreted bacterial proteins, and extracellular DNA (3).

Studies have shown that poly-*N*-acetylglucosamine (PNAG), also known as polysaccharide intercellular adhesion in Staphylococcus epidermidis, is the main exopolysaccharide in the S. aureus biofilm matrix (4). The products of the four genes in the *icaADBC* operon are required for the biosynthesis of PNAG (5). The fifth gene, intercellular adhesin locus regulator (*icaR*), is located upstream and is transcribed divergently from *icaADBC*. IcaR encodes a 186-amino-acid protein homologous to the transcriptional regulators of the TetR family (6). IcaR was reported to repress *icaADBC* transcription by binding to a 42-bp sequence within the *icaR-icaA* intergenic region (7). Additionally, previous reports have shown that several environmental factors, such as glucose, NaCl, and ethanol, influence biofilm elaboration by affecting *icaA* and *icaR* expression (8–11). Expression of *icaA* was unaffected by ethanol directly; however, it increased by repressing *icaR* transcription. Conversely, the induction of *icaA* expression by glucose or NaCl was *icaR* independent.

In previous studies (12, 13), JP080, previously named TF2758, was a superbiofilm-elaborating strain in which the *ica* operon and an unknown 7-gene cluster (*satf2580* to *satf2586*) showed high transcriptional expression levels in the comparative gene expression analysis of JP080 and the non-biofilm-elaborating strain ATCC 49775. A novel AcrR family regulator gene, designated regulator of biofilm (*rob*), was found in the 7-gene cluster. In the strain FK300, a *rsbU*-repaired derivative of S. aureus NCTC8325-4, Rob repressed biofilm elaboration through SAOUHSC_2898, a critical factor mediating biofilm elaboration and recognizing/binding to the 5-bp (TATTT) motif within the *icaR*-*icaA* intergenic region. The nonsense mutation in the *rob* gene of JP080 affected protein function and resulted in a marked increase in biofilm elaboration and PNAG production. Intriguingly, the complementation of JP080 with the wild-type *rob* did not completely abolish the superbiofilm-elaborating phenotype. We found another missense mutation in the biofilm-relevant gene *icaR*.

In this study, we examined the effects of *icaR* mutation on biofilm elaboration and explored why JP080 displayed a superbiofilm-elaborating phenotype with two mutations in *rob* and *icaR*.

## RESULTS

### Identification of a mutation in *icaR*.

**FIG 1:**
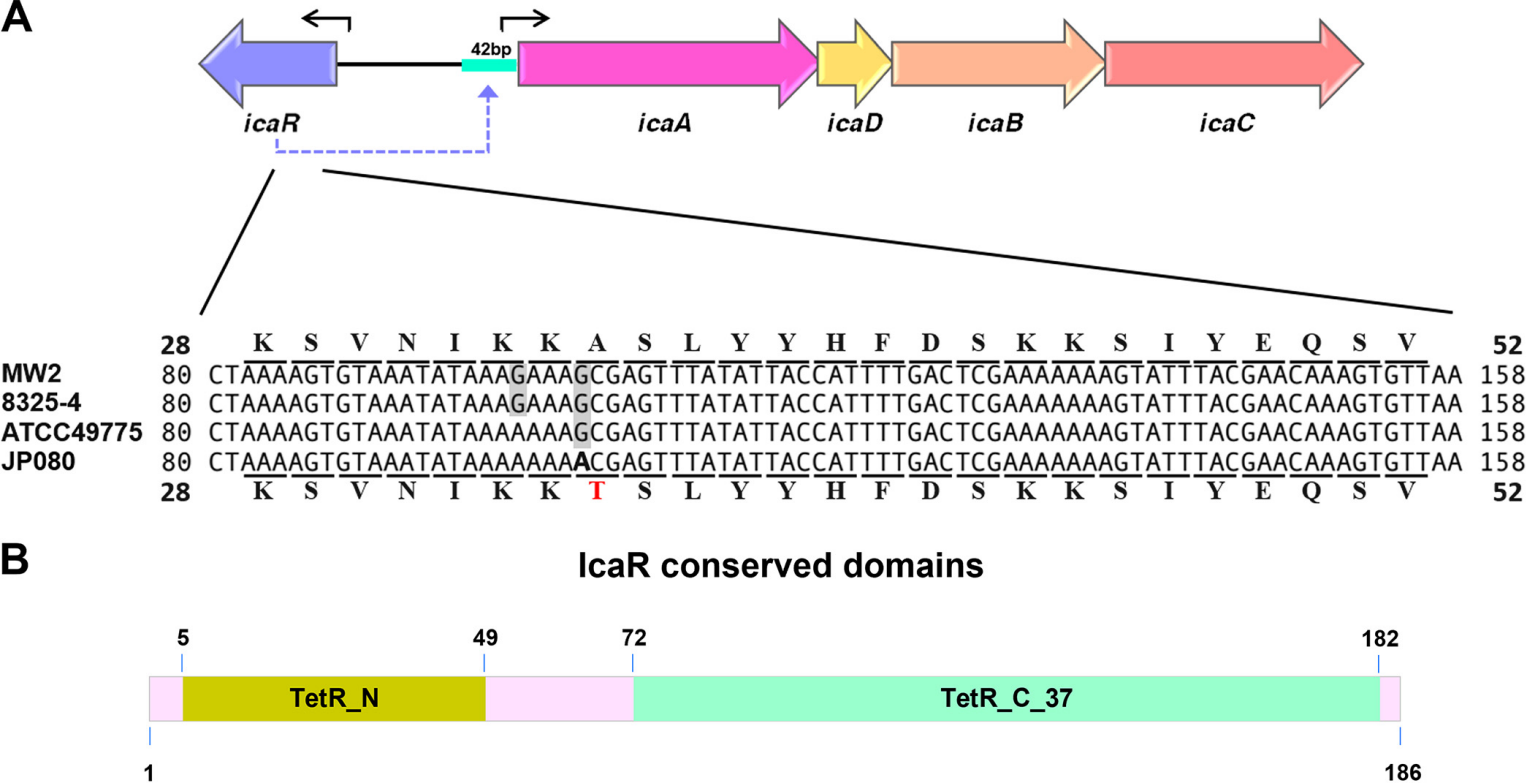
Identification of a missense mutation in the *icaR* gene of JP080 and the domain structure of its transcript. (A) Comparison of the nucleotide and amino acid sequences of the *icaR* gene in MW2, 8325-4, ATCC 49775, and JP080. The numbers shown on both sides are the nucleotide sequence and amino acid sequence positions in the open reading frame (ORF) of *icaR*. Altered amino acids (A to T) were indicated in red by the mutation at nucleotide position 103 (G to A). (B) Structural characteristics of IcaR. The TetR_N domain (amino acids 5 to 49) and the TetR_C_37 domain (amino acids 72 to 182), both of which are TetR-type helix-turn-helix (HTH) domains, which is a major structural motif capable of binding DNA, were detected in IcaR.

We had reported on superbiofilm-producing S. aureus JP080, previously named TF2758, a clinical isolate from atheroma (13). A nonsense mutation in the AcrR family regulator gene, *rob*, affected its function, resulting in increased biofilm elaboration in JP080. Intriguingly, the complementation of JP080 with wild-type *rob* did not completely abolish the superbiofilm-elaborating phenotype. Consequently, we obtained the complete genome sequence of JP080 (14) and searched for mutations or deletions in the gene(s) relevant to biofilm hyperproduction. We found another missense mutation at position 103 (G to A) in the *icaR* gene, characterized by an amino acid substitution of alanine by threonine in codon 35 (Ala35Thr) of the protein (Fig. 1A). Additionally, mutations of other regulatory factors in JP080 were investigated using the complete genome information of NCTC8325-4. However, no mutations were detected in other negative regulators of biofilm elaboration, such as *spx* and *tcaR*.

IcaR possesses regions homologous to those of the TetR family of transcriptional regulators (6). There are two conserved domains, the TetR_N domain (amino acids 5 to 49) and the TetR_C_37 domain (amino acids 72 to 182), both of which are TetR-type helix-turn-helix (HTH) domains, and Ala35 is located within the conserved domain TetR_N domain (Fig. 1B). The HTH is a major structural motif capable of binding DNA. Therefore, we hypothesized that the amino acid change might affect the function of IcaR, and we next investigated that by comparing the phenotypes of FK300 and its isogenic *icaR* single mutant (G103A in *icaR*) and *rob* and *icaR* double mutant (A331T in *rob* and G103A in *icaR*). The mutation A331T was the one originally identified in JP080.

### Effect of a missense mutation in *icaR* on biofilm elaboration and PNAG production.

**FIG 2:**
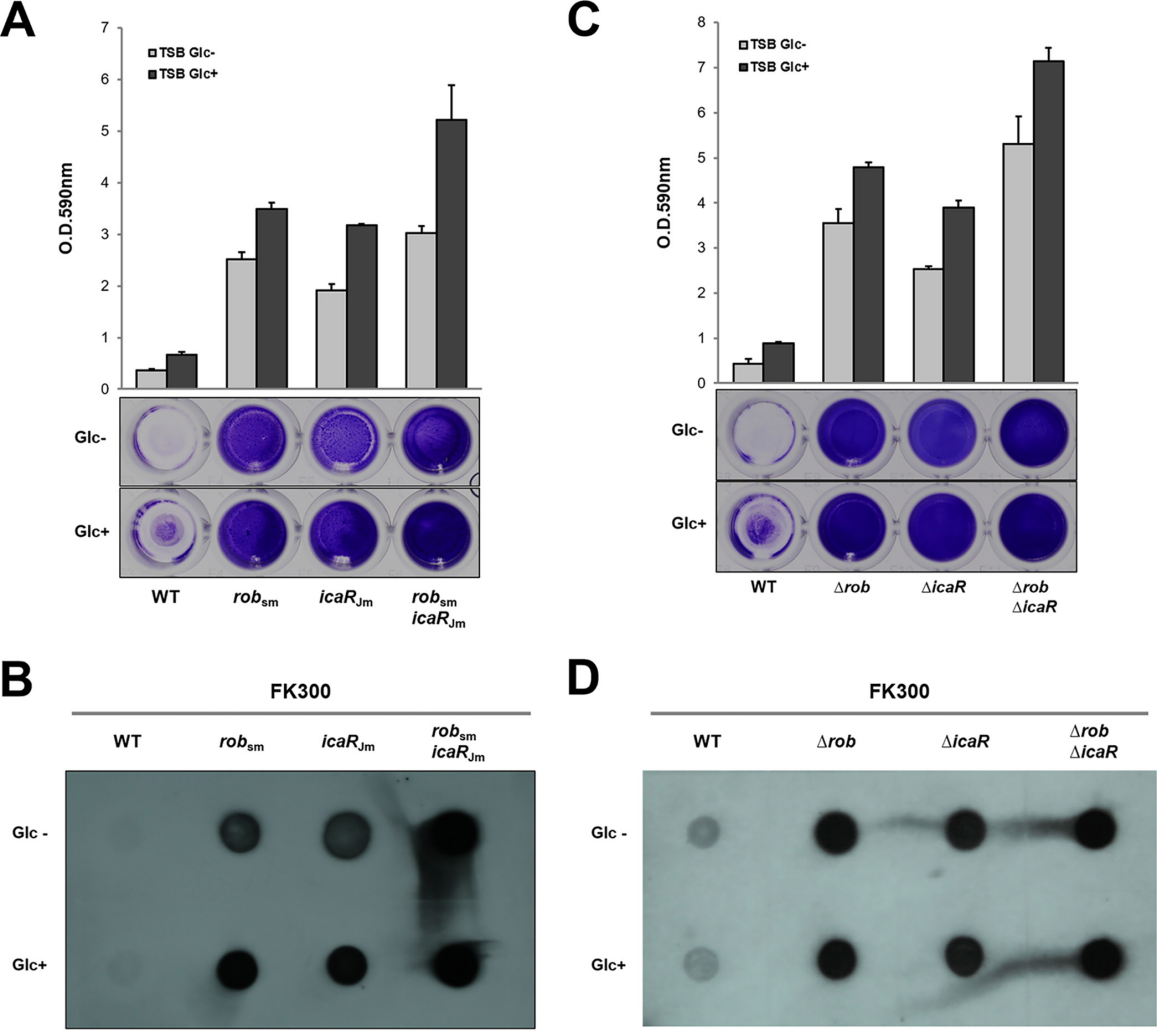
Introduction of A35T mutation in IcaR leads to increased biofilm elaboration and PNAG synthesis in strain FK300. Biofilm elaboration (A, C) and PNAG production of FK300 WT and its mutants (B, D). Bacteria were grown TSB in the presence (Glc^+^) or absence (Glc^−^) of 1% glucose. Biofilm elaboration was measured using the polystyrene microtiter plate assay described in the Materials and Methods section. The averages and standard errors from each sample are shown. Extracts from overnight cultures were spotted on a membrane, and PNAG was detected using rabbit antipolysaccharide intercellular adhesion, as described in the Materials and Methods section. *rob*_sm_, FK300 with an A331T mutation in *rob icaR*_Jm_, FK300 with a G103A mutation in *icaR*.

To determine whether the amino acid change (Ala35Thr) in IcaR affected biofilm elaboration, we reconstructed the mutation (*icaR*_Jm_:G103A in *icaR*) identified in JP080 using the standard strain FK300, a *rsbU*-repaired derivative of S. aureus NCTC8325-4, which produced almost no biofilm. As shown in Fig. 2A, the *rob* mutation (*rob*_sm_:A331T in *rob*) increased biofilm elaboration, as previously demonstrated (13). Similarly, the *icaR* mutation (G103A) enhanced its ability to elaborate biofilms. Moreover, we found that the double mutation of *rob* and *icaR* (*rob*_sm_ *icaR*_Jm_) in FK300 produced significantly more biofilms than that of wild type (WT) or single mutants (*rob*_sm_ and *icaR*_Jm_). Furthermore, we constructed *rob* and *icaR* deletion mutants and compared biofilm elaboration activities. As shown in Fig. 2C, the biofilm-elaborating abilities of Δ*rob*, Δ*icaR*, and Δ*rob* Δ*icaR* were similar to that of *rob*_sm_, *icaR*_Jm_, and *rob*_sm_ *icaR*_Jm_, respectively. Optical density values at 590 nm of FK300 *rob*_sm_ *icaR*_Jm_ and FK300 Δ*rob* Δ*icaR* were around 4.0 and 6.0, respectively, which is comparable or more than that of JP080, which is around 4.0 (13).

PNAG, the main component of staphylococcal biofilms, is synthesized by enzymes encoded by *icaADBC*, which are negatively regulated by *icaR*. To further examine the impact of the mutations on biofilm elaboration in FK300, we measured PNAG production in the WT strain and its derivatives (Fig. 2B and D). Consistent with the results of the biofilm assay, *rob*_sm_ *icaR*_Jm_ and Δ*rob* Δ*icaR* showed a much stronger PNAG-producing ability, while the mutation or deletion of *rob* or *icaR* increased PNAG. These results indicate that the missense mutation in *icaR* (G103A) enhanced biofilm elaboration and PNAG production of JP080. Therefore, besides the *rob* mutation, this could be another factor that causes biofilm elaboration in clinical isolates of S. aureus.

### Effect of missense mutations on *ica* operon expression in S. aureus strain FK300.

**FIG 3:**
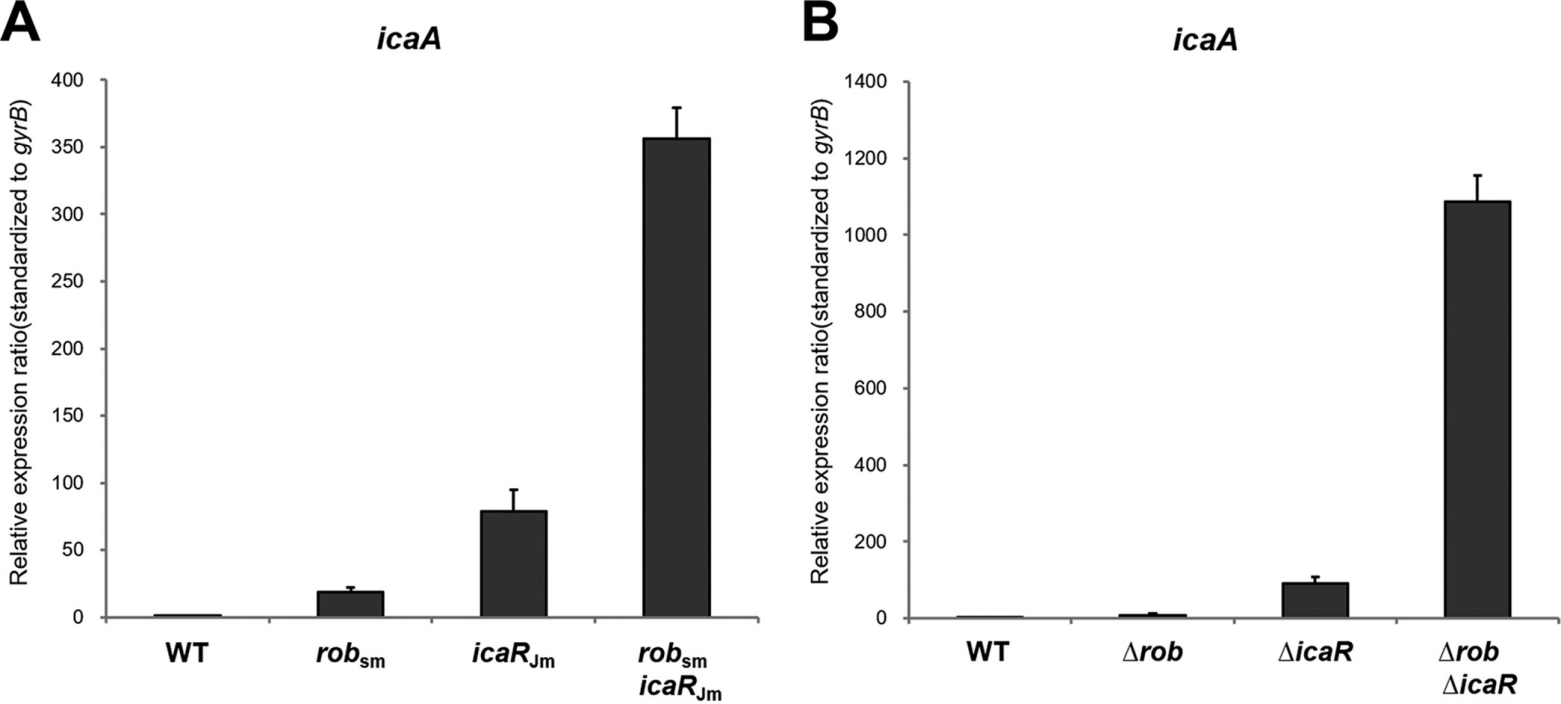
Effects of *icaR*, *rob* mutations, and *icaR*, *rob* deletions on *ica* operon expression in FK300. Quantitative measurements of *ica* operon transcription by qPCR in *icaR* and *rob* substitution mutants (A) and *icaR* and *rob* deletion mutants (B). Total RNA preparation, cDNA synthesis, and qPCR were performed as described in the Materials and Methods section. Transcript levels in *icaR*, *rob* substitution, and deletion mutants were determined in comparison with those in the wild-type strain FK300. The expression of the *gyrB* gene was used for sample normalization. Error bars indicate standard errors.

To confirm whether increased biofilm elaboration was associated with increased *ica* operon transcription, we measured *ica* operon transcriptional levels in these mutants by quantitative real-time RT-PCR (qRT-PCR) (Fig. 3). The results indicated that *icaR* deletion in FK300 increased *ica* operon expression 90-fold relative to that in the WT strain. Similarly, the *icaR* missense mutation in FK300 resulted in a 79-fold increase in *ica* operon transcription.

Notably, *rob*_sm_ *icaR*_Jm_ caused an approximately 350-fold increase in *ica* operon expression, and *rob* and *icaR* double deletion caused a >1,000-fold increase in *ica* operon expression compared with the expression in WT. Additionally, we found that a stop mutation in *rob* (A331T) identified in JP080 increased *ica* operon expression (Fig. 3A) and promoted PNAG production (Fig. 2B). These data further demonstrated that *rob* and *icaR* are two important factors controlling biofilm elaboration in FK300 and that the stop mutation in *rob* (*rob*_sm_:A331T) and the missense mutation in *icaR* (*icaR*_Jm_:G103A) were associated with the hyperproduction of PNAG and biofilm via an *ica*-dependent pathway in JP080.

### The missense mutation in *icaR* affects its ability to bind to the promoter of the *ica* operon.

**FIG 4:**
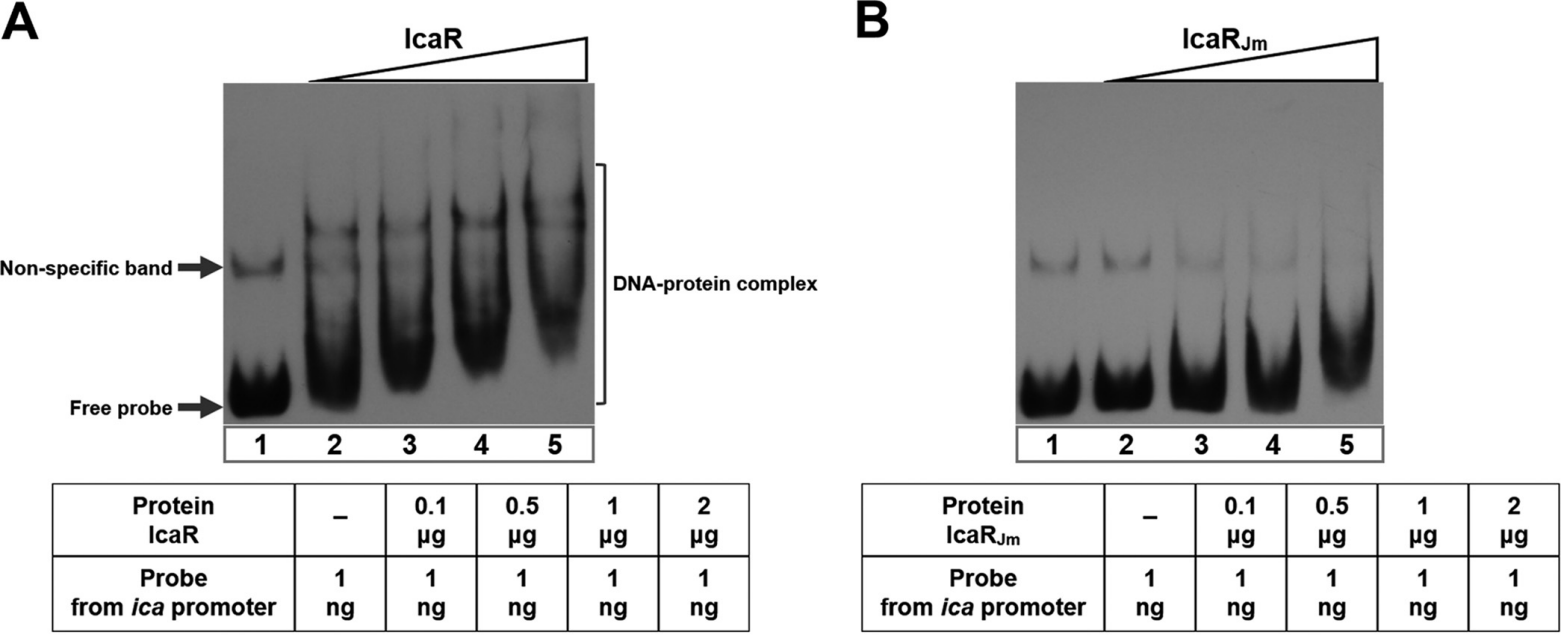
IcaR from JP080 is unable to bind to the *ica* promoter. EMSAs for the DNA-binding activity of IcaR (A) and IcaR_Jm_ (B) to the *icaR-icaA* intergenic region. EMSAs were performed in the absence (lane 1) or presence (lanes 2 to 5) of the purified proteins.

The protein encoded by *icaR* belongs to the TetR family of transcriptional regulators. It represses *icaADBC* transcription by binding to a region immediately upstream of the *icaA* start codon. To investigate whether the missense mutation affected IcaR function, we performed an electrophoretic mobility shift assay (EMSA). We purified FK300 IcaR and JP080 IcaR (IcaR_Jm_) proteins from Escherichia coli and analyzed their binding to an intergenic fragment between *icaR* and *icaA*. As shown in Fig. 4A, FK300 IcaR could bind to the *ica* promoter region. Furthermore, as the concentration of the IcaR protein increased, shifted bands were observed. However, IcaR_Jm_ failed to shift the DNA probe (Fig. 4B). These results demonstrated that IcaR_Jm_ was defective in its ability to bind to the *icaADBC* promoter, consistent with the biofilm assay and qRT-PCR data.

## DISCUSSION

The mutation we identified in the *icaR* of JP080 was located within the TetR DNA-binding domain. Therefore, we hypothesized that the A35T substitution affects the function of IcaR. Our data showed that the introduction of the A35T substitution in IcaR in the low biofilm-elaborating strain, FK300, increased biofilm elaboration and PNAG production. Additionally, *ica* operon expression increased in *icaR*_Jm_. A recent study reported that a V176E change in the TetR_C_37 domain of IcaR led to a significant increase in *icaADBC* operon transcription and PNAG production in the periprosthetic joint infected with the S. aureus strain (15). A single mutation in the functional domain of IcaR was considered to have a major effect on S. aureus biofilm formation capacity. Our study found that the IcaR protein in FK300 did bind to the *ica* promoter, consistent with a Jefferson et al. report (7). However, the IcaR protein in JP080 failed to bind to the *ica* promoter. These suggest that the alanine-to-threonine substitution at residue 35 (A35T) affected its function, enhancing *icaADBC* expression and subsequent biofilm elaboration and PNAG production.

**FIG 5:**
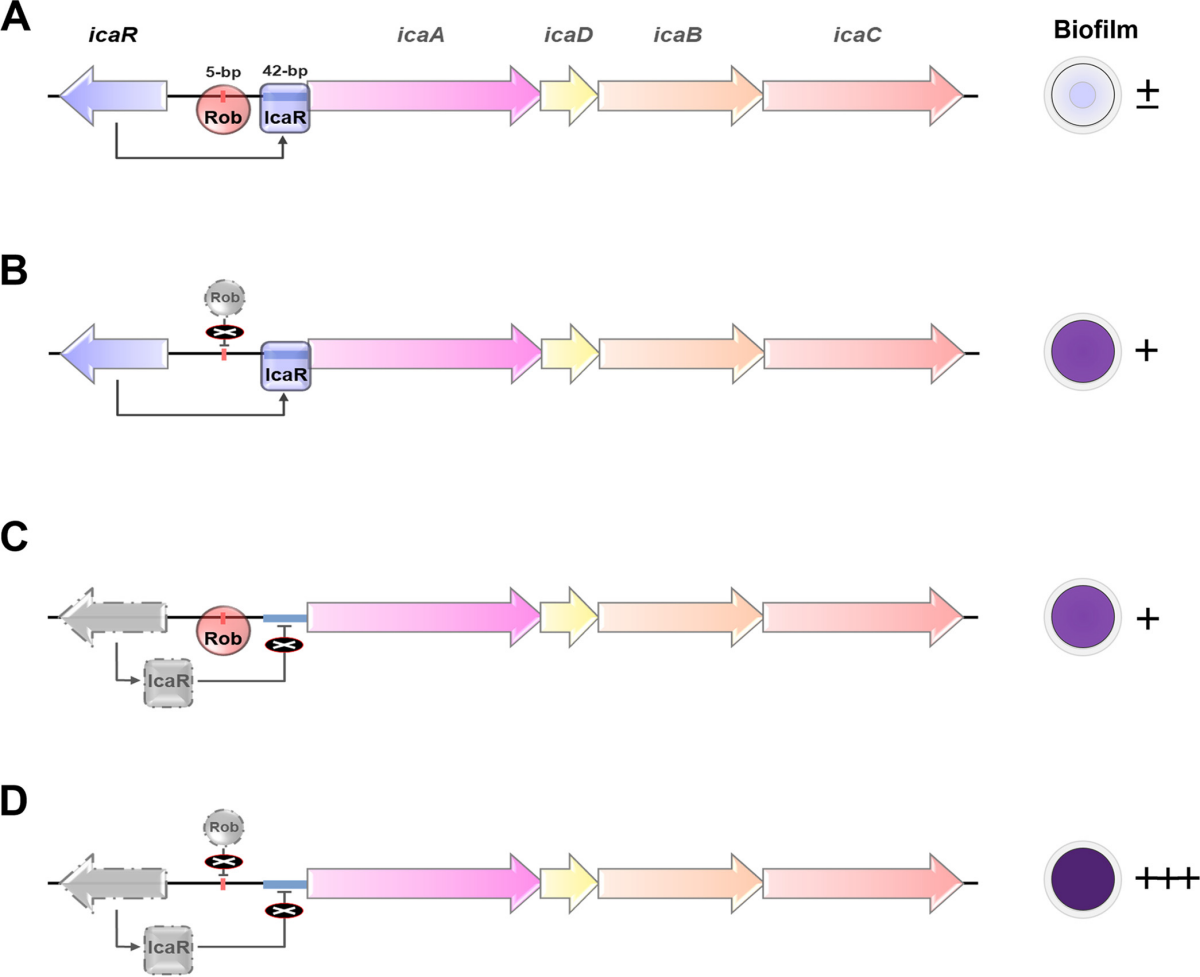
Proposed models of regulation of biofilm elaboration in S. aureus. (A) In S. aureus NCTC8325-4, *rob* and *icaR* are two transcriptional regulators. Rob recognizes the 5-bp (TATTT) motif within the *icaR*-*icaA* intergenic region (13). IcaR binds to a 42-bp region within the *ica* promoter region (7). No PNAG and biofilm are produced. (B) However, the nonsense mutation of *rob* affects its protein function in activating *icaR* expression and repression of *icaADBC* (13) and then trigger the production of PNAG and biofilm. (C) The missense mutation of *icaR* abolishes the binding ability of 42-bp region and contributes biofilm elaboration and PNAG production. (D) In S. aureus JP080, the mutations of *rob* and *icaR* affect the functions of both proteins, resulting in derepression of *icaADBC* that, in turn, would confer hyperproduction of PNAG and biofilm.

We previously reported that S. aureus JP080, a clinical isolate from an atheroma, overproduces biofilm and PNAG due to a nonsense mutation in the *rob* gene (13). Rob is an important regulator of biofilm elaboration through SAOUHSC_2898 in a glucose-independent pathway and recognizing/binding to the 5-bp (TATTT) motif within the *icaR*-*icaA* intergenic region. We demonstrated that another missense mutation in the *icaR* gene led to a nonfunctional protein that cannot bind to the promoter of the *ica* operon and also contributed to biofilm elaboration in JP080. Rob and IcaR are two negative regulators of biofilm elaboration in the S. aureus standard strain (e.g., NCTC8325-4). Our data suggest that the mutations of *rob* and *icaR* found in JP080 are associated with the hyperproduction of PNAG and biofilm, and the effects of the two mutations on *ica* operon expression are not additive but synergistic. A previous study demonstrated that the complementation of JP080 with a plasmid expressing *icaR* completely abolished biofilm elaboration (13). This means that the biofilm elaboration pathway by Rob also depends on *the ica* operon. A model of the activation process by *rob* and *icaR* inactivation is shown in Fig. 5. When *rob* and *icaR* are intact, the *ica* operon is suppressed because of their binding to the 5-bp (TATTT) motif and the 42 bp within the *icaR*-*icaA* intergenic region by Rob and IcaR, respectively, resulting in attenuated PNAG production (Fig. 5A). Introducing nonsense mutation in *rob* results in a loss of suppressor activity, which induces the transcriptional activation signal of *ica* operon; however, IcaR remains active in suppressing transcriptional activation of *the ica* operon (Fig. 5B). Therefore, activation of the *ica* operon may be limited. Similarly, when the mutation is introduced in *icaR*, suppression by IcaR is lost; however, intact Rob still suppresses the transcriptional activation signal of the *ica* operon (Fig. 5C). When double mutations are introduced into *rob* and *icaR*, full transcriptional activation of *ica* operon may occur because of no suppression by Rob or IcaR (Fig. 5D). This synergistic action of Rob and IcaR likely explains the superbiofilm elaboration in JP080.

Our previous study demonstrated that Rob could bind to the TATTT motif, which is different from the IcaR-binding site (42-bp region immediately upstream of the *icaA* start codon). It thus seems unlikely that Rob and IcaR compete to bind to the *ica* promoter (13). However, the *rob* deletion mutant decreased *icaR* expression. This further suggested that there should be a mechanism of indirect interaction of *icaR* and Rob. The detailed relationship between IcaR and Rob should be investigated in further studies, including the search for potential competition for ica promoter binding.

Elaboration of biofilms appears to be a survival strategy for bacteria. If a staphylococcal biofilm is established, it becomes difficult to eliminate. Biofilm elaboration in living tissues or indwelling medical devices increases bacteria’s resistance to antibiotics and the immune system (16–18). Kahl’s team previously reported that the clinical S. aureus, isolated from patients with cystic fibrosis, presented a mucoid phenotype because of the 5-bp deletion within the *icaR*-*icaA* intergenic region and showed more resistance to important antistaphylococcal antibiotics than nonmucoid isolates (19, 20). This strategy makes it difficult to treat chronic biofilm-associated infections. Previous studies on the mechanism of biofilm formation of staphylococci have been conducted using S. epidermidis, which is generally regarded as a strong biofilm producer. In the case of S. aureus, biofilm-producing ability is variable according to strains. S. aureus often causes acute infection with dissemination of bacteria inside the body, and biofilm formation may be a negative factor for virulence. The complex regulation mechanism found in S. aureus in this study might have been developed owing to the necessity of S. aureus to behave as a multipotential pathogen in acute and chronic settings. A more thorough understanding of biofilm mechanisms is essential for controlling and eradicating biofilm-related infections. Numerous staphylococcal regulatory factors, such as Rbf, Rob, SarA, SrrAB, TcaR, and IcaR, reportedly affect biofilm elaboration by controlling *ica* operon expression (8, 13, 21–26). Our study provides additional insight into understanding the regulatory mechanism of biofilm elaboration, but further study is necessary to uncover the whole picture of this mechanism in S. aureus.

## MATERIALS AND METHODS

### Bacterial strains and growth media.

The bacterial strains and plasmids used are listed in Table 1. S. aureus strain JP080, previously named TF2758, is a clinical isolate from a patient with atheroma in Japan. S. aureus FK300, a *rsbU*-repaired derivative of strain NCTC8325-4, was used to study the effect of *icaR* mutation on biofilm development. The restriction-defective S. aureus strain RN4220 (27) was used as the initial recipient for electroporation of the recombinant plasmids. S. aureus strains were routinely grown in tryptic soy broth (TSB) (Becton, Dickinson Microbiology Systems, Cockeysville, MD) or tryptic soy agar (TSA) plates. Tetracycline was added when necessary at a final concentration of 5 μg/mL. The Escherichia coli strain DH5α was used for plasmid construction and maintenance. E. coli was grown in Luria-Bertani (LB) broth (5 g yeast extract, 10 g polypeptone, and 10 g NaCl per L, pH 7.2) or on LB agar. When required, antibiotics were added to the culture medium at final concentrations of 100 μg/mL for ampicillin and 10 μg/mL for tetracycline.

**TABLE 1:** Strains and plasmids used in the present study

Strain or plasmid	Relevant characteristic(s)	Source or reference
S. aureus strains		
JP080	Wild-type clinical isolate, biofilm positive	14
FK300	Derivative of NCTC8325-4 (*rsbU* repaired)	13
RN4220	Restriction-negative strain, NCTC8325-4 derivative	27
*icaR*_Jm_	FK300 with a missense mutation (G103A) in *icaR*	This study
*rob*_sm_	FK300 with a stop mutation (A331T) in *rob*	13
*rob*_sm_ *icaR*_Jm_	FK300 with a stop mutation in *rob* and a missense mutation in *icaR*	This study
△*rob*	FK300 △*rob*	13
△*icaR*	FK300 △*icaR*	This study
△*rob* △*icaR*	FK300 △*rob* △*icaR*	This study
E. coli strains		
DH5α	Cloning strain	TaKaRa
BL21(DE3)	Host for recombinant protein production	Novagen
Plasmids		
pKFT	Vector for allele replacement	28
pET-22b(+)	E. coli expression plasmid	Novagen
pET22b-*icaR*	His-IcaR expression plasmid	13
pET22b-*icaR*_Jm_	His-IcaR_Jm_ expression plasmid	This study

### Plasmid and strain construction.

We used the E. coli-S. aureus shuttle vector pKFT (28) to construct FK300 mutants. Routine DNA manipulations were performed as previously described (13). The oligonucleotide primers used for PCR are listed in Table 2. The recombinant plasmids were first transformed into DNA restriction system-deficient S. aureus RN4220 and then into strain FK300 or its derivatives. Allele replacement was performed as described by Kato et al. (28). Markerless deletion mutants were screened by PCR, and fragments were confirmed by DNA sequencing using the BigDye Terminator v3.1 cycle sequencing kit (Applied Biosystems, USA).

**TABLE 2:** Primers used in the present study

Primer	Sequence (5′–3′)
Primers used for plasmid and strain construction	
*rob*_sm_-1	ACAACGCCCTTAATTGTTGCC
*rob*_sm_-2	GCAACAATTAAGGGCGTTGTTACCAAAG
*rob*-1	TACCAAGCTTCCTCTAACAACTGTTTTAC
*rob*-2	CATCAACTAGTTTGTGCGCTATTTCTTC
*rob*-3	GCTGTTGCAATCATTATCAACTAGTG
*rob*-4	AGGTAAAGCTTTAGCGTATTGTAGCG
*icaR*-1	TGGTGAAGCTTGATCAACGATAGTATC
*icaR*-2	GTAGCGAATACACTTCATCTACCGTCATACCCCTTCTCTG
*icaR*-3	CAGAGAAGGGGTATGACGGTAGATGAAGTGTATTCGCTAC
*icaR*-4	TAATAAAGCTTGATACCATCGTACTC
*icaR*_Jm_-1	TTTATATTACCATTTTGACTCG
*icaR*_Jm_-2	CTCGTTTTCTTTATATTTACAC
Primers used for qPCR	
*gyrB* for	AGGTCTTGGAGAAATGAATG
*gyrB* rev	CAAATGTTTGGTCCGCTT
*icaA* for	AGTTGTCGACGTTGGCTAC
*icaA* rev	CCAAAGACCTCCCAATGT
Primers used for EMSA	
*ica*-p-F	ATTGCGTTATCAATAATCTTATCCTTC
*ica*-p-R (5-Biotin)	TTGCAATTCCTTTACCTACCTTTC
pET-22b-IcaR-F	GGAATTCCATATGCACCACCACCACCACCACTTGAAGGAT AAGATTATTGATAACGC
pET-22b-IcaR-R	CCCAAGCTTTTATTTCTTCAAAAATATATTTAGTAGCG

### Biofilm assay.

We conducted biofilm assays using flat-bottom 96-well polystyrene plates (TrueLine; Nippon Genetics Co., Ltd., Japan) as previously described (29). Diluted overnight cultures of each strain were cultured in TSB or TSB with 1% glucose, followed by incubation at 37°C for 24 h. The biofilms were washed thrice with sterile phosphate-buffered saline (PBS) (137 mM NaCl, 2.7 mM KCl, 10 mM Na_2_HPO_4_12H_2_O, and 1.8 mM KH_2_PO_4_, pH 7.4) and stained with 1% crystal violet for 15 min. The wells were washed in a container by immersing and agitating gently 10 times in tap water to remove unbound crystal violet. Last, the biofilm-bound crystal violet was dissolved in 33% glacial acetic acid, and absorbance was measured at 590 nm using an Immuno-Mini NJ-2300 spectrophotometer (Nalge Nunc International K.K., Tokyo, Japan).

### PNAG detection.

PNAG production in S. aureus FK300 and its mutants was detected as previously described (5). The cells grown in a TSB medium with glucose (Glc^+^) or without glucose (Glc^−^) were harvested and resuspended in 50 μL of 0.5 M EDTA (pH 8.0). After incubation at 100°C for 5 min, the cells were removed by centrifugation, and 40 μL of the supernatant was incubated with 10 μL of proteinase K (20 mg/mL; Nacalai Tesque, Inc., Kyoto, Japan) at 37°C for 30 min. The extracts were then immobilized on a nitrocellulose membrane (Amersham Protran NC 0.45; GE Healthcare, Buckinghamshire, UK), dried, and blocked with 5% skim milk in PBS containing 0.1% Tween 20. After 2 h incubation with 1:10,000 rabbit anti-PNAG antiserum (30), bound antibodies were detected with 1:10,000 peroxidase-conjugated goat anti-rabbit immunoglobulin G (IgG) antibodies (MP Biomedicals, LLC-Cappel Products, OH, USA) and developed using Pierce ECL Western blotting substrate (Thermo Scientific, Rockford, IL, USA).

### RNA isolation, reverse transcription, and real-time PCR.

We prepared total S. aureus RNA using the FastRNA Pro Blue kit (MP Biomedicals, LLC, Santa Ana, CA, USA), according to the manufacturer’s instructions. RNAs were treated with RQ1 RNase-free DNase (Promega, Madison, WI, USA) for 30 min at 37°C and then reverse transcribed using a Transcriptor first strand cDNA synthesis kit (Roche, Mannheim, Germany). To measure *ica* operon expression, quantitative real-time RT-PCR (qRT-PCR) was performed using the SsoAdvanced Universal SYBR green supermix (Bio-Rad, Hercules, CA, USA) and a CFX96 real-time PCR detection system (Bio-Rad). All PCR runs were performed in triplicate, and the data were analyzed using CFX Manager software (version 3.0; Bio-Rad) according to the manufacturer’s instructions. The housekeeping gene, gyrase subunit B (*gyrB*), was used as a reference gene to normalize the expression level of the target gene in each reaction.

### Protein purification.

Recombinant His-tagged IcaR and IcaR_Jm_ were purified as described by Jeng et al. (6) with a few modifications. The *icaR* gene was amplified by PCR from the genomic DNA of FK300 or JP080 using primers pET-22b-IcaR-F and pET-22b-IcaR-R (Table 2) and cloned into the expression vector pET-22b(+) (Novagen). E. coli BL21(DE3) cells, transformed with the recombinant plasmid, were grown in 300 mL LB medium containing 100 μg/mL ampicillin at 37°C to an optical density of 0.5 at 600 nm. Protein expression was induced with 0.5 mM isopropyl-β-d-thiogalactopyranoside (Nacalai Tesque, Inc., Kyoto, Japan), and cells were harvested after incubation at 30°C for 2 days. Subsequently, the harvested cells were lysed by sonication on ice. His_6_-tagged IcaR and IcaR_Jm_ were purified from the supernatant using Talon metal affinity resins (Clontech Laboratories, Inc.) according to the manufacturer’s protocol. Protein expression and purity were analyzed by sodium dodecyl sulfate-polyacrylamide gel electrophoresis on a 12% gel. Protein concentrations were measured using the Bio-Rad protein assay (Bio-Rad, Hercules, CA), with bovine serum albumin as the standard.

### EMSA.

Gel shift assays were performed as described previously to determine whether recombinant IcaR and IcaR_Jm_ bind to the *ica* promoter region (7). A 198-bp DNA fragment containing the *icaR-icaA* intergenic region was amplified by PCR using the primers listed in Table 2. After, the PCR products were purified using a QIAquick gel extraction kit (Qiagen). Binding reactions were performed by adding 0.1 to 2 μg of purified recombinant protein and 1 ng of the biotin-labeled probe. The reaction mixtures were incubated for 20 min at room temperature and then electrophoresed on a 5% polyacrylamide gel in prechilled 1× Tris-borate-EDTA buffer. DNA was then transferred onto a nylon membrane (BioDyne B; Pall, USA), and band shifts were detected by exposing the dried membranes to X-ray films.
